# Seizure-like movements caused by residual sevoflurane inside the anesthesia machine: A case report

**DOI:** 10.1097/MD.0000000000024495

**Published:** 2021-01-29

**Authors:** Jianqiao Zheng, Li Du, Lu Zhang

**Affiliations:** a Department of Anesthesiology, West China Hospital of Sichuan University; b Department of Anesthesiology, Sichuan Cancer Center, School of Medicine, Sichuan Cancer Hospital & Institute, University of Electronic Science and Technology of China; c Department of Anesthesiology, West China Hospital of Sichuan University, Chengdu, Sichuan, China.

**Keywords:** anesthesia equipment, anesthetics inhalation, checkout, convulsions, sevoflurane

## Abstract

**Rationale:**

Sevoflurane-induced seizures are most often caused by high concentrations of sevoflurane during anesthesia induction. However, in this case, we found a rare case of seizure-like movements caused by residual sevoflurane inside the anesthesia machine. Therefore, we propose that the detection of residual anesthesia-inhaled drugs should be included in pre-anesthesia checkout procedures.

**Patient concerns:**

An 11-year-old girl with a history of epilepsy was scheduled for emergency appendectomy under general anesthesia. The patient presented with seizure-like movements caused by residual sevoflurane inside the anesthesia machine after pre-oxygenation during rapid sequence induction.

**Diagnoses:**

Based on the clinical presentation and previous history of seizures, sevoflurane-induced seizures were diagnosed.

**Interventions:**

A washout procedure was performed by turning the oxygen flow up to 10L/min to wash out the residual sevoflurane from the anesthesia machine.

**Outcomes:**

The seizures ceased spontaneously, and the vital signs of the patient were stable during the washout procedure. Rapid sequence anesthesia induction and total intravenous anesthesia maintenance were uneventful. Surgery was performed as planned, and there were no postoperative problems. The patient was discharged after 4 days without complications and was well on follow-up.

**Lessons:**

The check-up procedure of residual anesthesia-inhaled drugs inside the anesthesia machine should be included in the checkout design guidelines, or else the washout procedure should be performed in the pre-anesthesia checkout procedures.

## 1 Introduction

Pre-anesthesia checkout procedures recommended by the Sub-Committee of the American Society of Anesthesiologists Committee on Equipment and Facilities help to detect the problems of the anesthesia machine preoperatively.^[1]^ Improperly checking anesthesia equipment prior to use can lead to patient injury and has also been associated with an increased risk of severe postoperative morbidity and mortality.^[2,3]^ Anesthesiologists should check the anesthesia equipment according to the guidelines of the pre-anesthesia checkout procedures. In most situations, residual anesthesia-inhaled drugs inside anesthesia machines can be neglected, and the check-up of residual anesthesia-inhaled drugs is not recommended in the guidelines.^[1,4]^ the residual inhaled drugs could reach a high concentration, which could induce clinical complications in special situations, such as patients with a history or family history of malignant hyperthermia (MH).^[5]^

Sevoflurane-induced seizures are most often caused by high concentrations of sevoflurane combined with alveolar hyperventilation during mask induction of anesthesia.^[6,7]^ Here, we report a rare case of seizure-like movements caused by residual sevoflurane inside an anesthesia machine after pre-oxygenation during rapid sequence anesthesia induction in a girl with a family history of epilepsy. We propose that residual anesthesia-inhaled drugs in anesthesia machines could be included in the pre-anesthesia checklist, especially when patients have contraindications for the use of inhalation anesthesia, such as MH and epilepsy.

This study was approved by the Institutional Review Board of the West China Hospital of Sichuan University. Consent was obtained from the patients’ parents prior to submission of this case for publication.

## 2 Case report

An 11-year-old, 135 cm, 30-kg girl presented with pain in the right iliac region with fever and vomiting. Acute appendicitis was diagnosed on physical examination and abdominal ultrasound, and emergency appendectomy was scheduled. The patient had no previous experience of anesthesia, but she had a history of epilepsy. With oral valproate antiepileptic therapy, epilepsy symptoms have not occurred in recent years. Rapid sequence anesthesia induction and endotracheal intubation were selected according to the anesthesia standard for emergency surgery. The anesthesia machine was checked as a pre-anesthesia checklist by an anesthesiologist, and standard monitoring was applied when the patient arrived in the operating room. Pre-oxygenation was performed with a standard face mask attached to the circuit of the Datex-Ohmeda Smart Vent (Ohmeda 7900, Datex-Ohmeda, Madison, WI) at a fresh oxygen flow of 6L/min. No drugs were used during the pre-oxygenation process, and the SpO_2_ increased from 95% in room air to 100%. After 4 minutes of pre-oxygenation, the patient presented with symmetrical seizure-like movements of the upper extremities. We found that the expiratory sevoflurane concentration was as high as 3% on the monitor. Therefore, we checked the breathing circuit, vaporizer, and carbon dioxide absorbent, and found no problems and the vaporizer was closed during the pre-oxygenation procedure. We confirmed that sevoflurane in the breathing circuit was residual sevoflurane inside the anesthesia machine, and the convulsions were caused by residual sevoflurane. We used a new face mask and a bag valve device with a bag reservoir instead of an anesthesia machine for oxygen supply. Immediately, we turn up the oxygen flow to 10L/min in order to wash out the residual sevoflurane. The convulsions ceased spontaneously without treatment. The patient had no obvious discomfort, and his vital signs were stable. When the expiratory sevoflurane concentration decreased to zero on the monitor, a new breathing circuit was used for anesthesia induction. Rapid sequence anesthesia induction and endotracheal intubation were performed smoothly. Considering the contraindication for the use of inhalation anesthesia, total intravenous anesthesia was used during the operation, and anesthesia was uneventful. Surgery was performed as planned, and there were no postoperative problems. The patient was discharged after 4 days without complications and was well on follow-up.

## 3 Discussion

Despite the frequent use of sevoflurane in anesthesia without adverse reactions, there have been several reports of sevoflurane-induced seizures in patients anesthetized with sevoflurane.^[8,9]^ In these cases, the end-tidal concentration of sevoflurane ranged from 0.75% to 2.5%. Similar to our case, most previous reports of seizure-like activity during sevoflurane were characterized by symmetrical tonic-clonic motor activity, and no serious neurological adverse effects were found in the postoperative period. The mechanisms responsible for seizure-like activity following sevoflurane anesthesia remain to be defined. The action of sevoflurane at γ-aminobutyric acid-mediated receptors could induce interictal epileptiform discharges or seizures in children.^[10–12]^ Gibert et al demonstrated that the occurrence of major epileptiform signs (MES) is highly dependent on the sevoflurane concentration.^[13]^ The minimal alveolar concentration (MAC) of sevoflurane causing MES, calculated in 100% oxygen, corresponded to 1.75 surgical MAC.^[13]^ When the residual sevoflurane concentration approached 1.75, surgical MAC during pre-oxygenation, the occurrence of MES seems to be highly probable.

The monitoring of residual anesthesia-inhaled drugs inside the anesthesia machine is not included in the pre-anesthesia checkout procedures, only when the patients have contraindications for the use of inhalation anesthesia, such as those susceptible to MH. In patients susceptible to MH, residual halogenated anesthetics are required to wash out completely. However, in other situations, such as patients with epilepsy or a family history of epilepsy, the residual inhaled drugs were not asked to wash out completely.^[13]^ However, in special situations, the residual halogenated anesthetics could reach a high concentration, which could induce clinical complications. Therefore, we propose that the check-up of residual anesthesia-inhaled drugs inside the anesthesia machine could be included in the pre-anesthesia checklist.

In MH-susceptible patients, less than 5 parts per million (0.0005%) concentration of the anesthetic agent are known to be well tolerated.^[14]^ Recommendations of the washout time for preparing different anesthesia machines for MH-susceptible patients ranges from 15 to 70 minutes.^[15]^ Instructions of the washout time were at least 20 minutes with a fresh gas flow rate of more than 10 L/min.^[16,17]^ So, we can do this wash out procedure during the pre-anesthesia checkout procedures routinely.

## 4 Conclusion

Although clinical complications caused by residual anesthesia, inhaled drugs inside the anesthesia machine are rare, and no comprehensive clinical studies have been conducted to date. More attention should be focused on the pre-anesthesia checkout procedures of residual anesthesia-inhaled drugs, especially in patients who have contraindications for the use of inhalation anesthesia. We wish to propose that the check-up of residual anesthesia-inhaled drugs inside anesthesia machines should be included in the checkout design guidelines, and that the washout procedure should be routinely performed in the pre-anesthesia checkout procedures.

## Author contributions

**Data curation:** Jianqiao Zheng, Li Du.

**Supervision:** Jianqiao Zheng, Lu Zhang.

**Writing – original draft:** Jianqiao Zheng, Li Du.

**Writing – review & editing:** Jianqiao Zheng, Lu Zhang.

## Abbreviations

Abbreviations: MAC = minimal alveolar concentration, MES = major epileptiform sign, MH = malignant hyperthermia.
